# Natural Polyphenols and Mesenchymal Stem Cells: A New Insight in Bone Regenerative Medicine

**DOI:** 10.1155/sci/8019494

**Published:** 2025-09-12

**Authors:** Mohammad-Sadegh Lotfi, Fatemeh B. Rassouli

**Affiliations:** ^1^Novel Diagnostics and Therapeutics Research Group, Institute of Biotechnology, Ferdowsi University of Mashhad, Mashhad, Iran; ^2^Department of Biology, Faculty of Science, Ferdowsi University of Mashhad, Mashhad, Iran

**Keywords:** bone regeneration, mesenchymal stem cells, natural polyphenols, osteogenic differentiation

## Abstract

Bone defects pose significant clinical challenges, necessitating the development of innovative strategies to effectively restore damaged bone and recover normal function. Mesenchymal stem cells (MSCs) have emerged as a promising tool for bone regeneration due to their accessibility from various sources, ease of isolation and expansion, and intrinsic ability to differentiate into osteogenic lineages with minimal ethical concerns. However, successful bone repair using MSCs requires the incorporation of biocompatible osteoinductive agents, preferably derived from natural sources. Natural polyphenols, particularly flavonoids, exhibit potent pharmaceutical properties that modulate MSC fate toward osteogenic differentiation. These secondary metabolites promote osteogenesis by interacting with key bone regulatory signaling pathways, including bone morphogenetic protein 2 (BMP2)/SMAD, wingless-related integration site (Wnt)/β-catenin, nuclear factor kappa-light-chain-enhancer of activated B cell (NF-κB), and mitogen-activated protein kinase (MAPK). Beyond their osteoinductive capacity, flavonoids possess anti-inflammatory, antibacterial, and pro-angiogenic effects, which synergistically enhance bone formation both in vitro and in vivo, thereby amplifying their therapeutic potential. This review synthesizes current insights into the interaction between MSCs and natural flavonoids, detailing the molecular mechanisms driving their synergistic effects. It also highlights recent advancements in nanoformulation-based delivery systems aimed at addressing challenges like poor solubility and bioavailability. Although preclinical data strongly support the bone-protective properties of these agents, their clinical translation remains forthcoming. Future studies must focus on optimizing delivery methods, ensuring long-term safety, and rigorously validating therapeutic efficacy across various bone disorders.

## 1. Introduction

Compromised bone integrity, frequently caused by trauma, inflammation, chemotherapy, and age-related factors such as menopause, can lead to significant morbidity and disability if left untreated. Therefore, it is essential to develop effective approaches to restore bone health and improve quality of life. Stem cell therapy holds great promise for treating bone deficits, and mesenchymal stem cells (MSCs) are among the most commonly used options in preclinical and clinical practice. Several advantages contribute to the widespread use of MSCs, including their accessibility from various sources, such as bone marrow, adipose tissue, umbilical cord blood, peripheral blood, placenta, and Wharton's jelly [1–4]. In addition, the isolation of MSCs is relatively straightforward; they can be cultivated in large quantities, while maintaining genomic stability, and they have the ability to differentiate into chondroblasts and osteoblasts. Finally, yet importantly, applications of MSCs are associated with limited ethical concerns compared to other stem cell sources [5–7]. Effective bone regeneration using MSCs relies on osteoinductive molecules and osteoconductive scaffolds that act in a mechanically stable environment [8–12]. Therefore, osteogenesis induction of bone marrow MSCs (BM-MSCs) using natural agents loaded in biocompatible scaffolds holds great promise in promoting bone tissue engineering and repair.

Polyphenols constitute a diverse class of naturally occurring organic compounds characterized by multiple phenolic structures—aromatic rings bearing two or more hydroxyl groups. These bioactive molecules are abundantly present in various parts of plants, with particularly high concentrations in the peels and seeds of fruits. Their natural abundance, ease of extraction, and low cytotoxicity make polyphenols highly attractive candidates for biomedical applications. Extensive research has demonstrated that polyphenols exhibit a wide range of biological activities, including potent antibacterial, antioxidant, anti-inflammatory, and anticancer effects, highlighting their significant therapeutic potential [13–15].

Primarily, polyphenols are renowned for their strong antioxidant capacity. This property arises from their chemical structure, which enables them to donate electrons and neutralize harmful free radicals, thereby protecting cells from oxidative stress and damage. In bone tissue engineering, the antioxidant function of polyphenols is especially critical, as oxidative stress represents a major barrier to effective tissue repair and regeneration. By reducing oxidative damage, polyphenols help establish a more favorable microenvironment that promotes cell survival, proliferation, and differentiation. Importantly, the beneficial effects of polyphenols extend well beyond their antioxidant activity. For instance, polyphenols have a high affinity for proteins, allowing them to interact with various cellular receptors involved in bone regeneration. These interactions enable polyphenols to modulate key cellular signaling pathways, regulate enzyme activities, and influence gene expression, thereby affecting numerous physiological processes. Additionally, polyphenols possess antimicrobial properties, which can be instrumental in preventing infections during tissue repair, further supporting their application in bone regenerative medicine [16–18].

**Figure 1:**
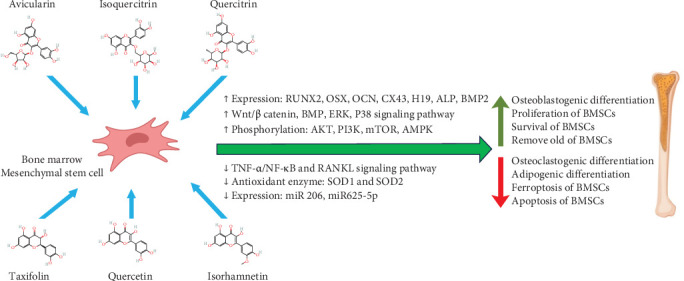
Effects of natural flavonoids on bone marrow derived MSCSs for bone regeneration.

Flavonoids are natural polyphenols found in dietary plants with valuable pharmacological properties. These secondary metabolites are categorized into bioflavonoids, isoflavonoids, and neoflavonoids, which differ in the degree of unsaturation, hydroxylation, oxidation, and glycosylation [19, 20]. Bioflavonoids are the most prevalent and widespread flavonoids in nature, among which quercetin, kaempferol, fisetin, isorhamnetin, and myricetin are prominent in the human diet [21]. Natural polyphenols have the potential to induce the differentiation and proliferation of osteoblasts while preventing osteoclast formation. As presented in Figure 1, the osteogenesis-inducing effects of flavonoids are mediated by their interaction with key bone signaling pathways, including the bone morphogenetic protein 2/SMAD (BMP2/SMAD) pathway, wingless-related integration site (Wnt)/β-catenin, nuclear factor kappa-light-chain-enhancer of activated B cells (NF-κBs) signaling, and mitogen-activated protein kinase (MAPK) signaling [10–18]. In addition, the anti-inflammatory, antibacterial, and angiogenesis-inducing activities of flavonoids enhance their ability to induce osteogenesis in vitro and in vivo, thereby amplifying their therapeutic potential.

Due to the pivotal role of MSCs in bone tissue regeneration and the significant impact of natural flavonoids on osteogenesis, this review offers a comprehensive and up-to-date perspective on the application of quercetin and structurally similar flavonoids in the osteogenic differentiation of MSCs. By elucidating the intricate interplay between these two key elements, we aim to provide a foundation for the development of innovative approaches in the field of bone regenerative medicine.

## 2. Methods

### 2.1. Search Strategy and Information Sources

We performed a thorough and systematic search across several electronic databases, including PubMed, Google Scholar, and Web of Science, to identify pertinent studies examining the potential of natural flavonoids in bone regeneration, with a particular emphasis on their effects on MSCs. The search employed a combination of keywords designed to capture relevant literature, specifically: (“Natural polyphenol” AND “Bone regeneration” AND “Mesenchymal stem cells”) [Title/Abstract]; (“Natural flavonoids” AND “Osteogenesis” AND “Stem cells”) [Title/Abstract]; (“Quercetin” AND “Osteogenic differentiation” AND “Mesenchymal stem cells”) [Title/Abstract].

### 2.2. Inclusion Criteria and Screening

Given the large volume of studies on quercetin, inclusion criteria were limited to original research articles investigating the osteogenic potential of quercetin and structurally related flavonoids, either alone or combined with other compounds, in vitro or in vivo. We excluded review articles, conference abstracts, and studies not directly addressing flavonoid-induced osteogenesis in MSCs or related bone regeneration models.

**Figure 2:**
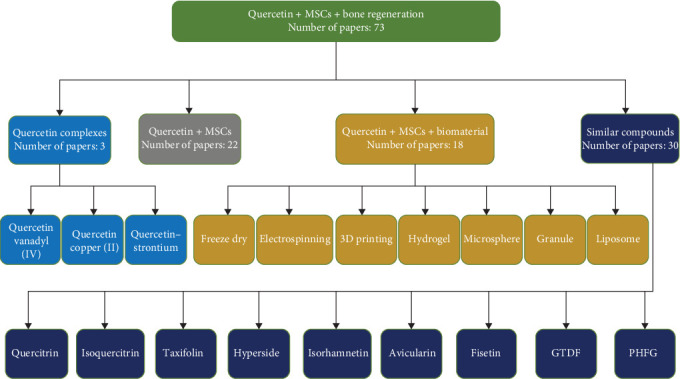
The search strategy and database flowchart.

As shown in Figure 2, our comprehensive search identified 73 original research papers that were screened for relevance. These were categorized as follows: 22 studies examined the direct effects of quercetin on MSC osteogenic differentiation; three studies focused on quercetin complexes and their role in bone regeneration; 18 studies explored biomaterials incorporating quercetin for bone tissue engineering applications; and 30 studies evaluated natural flavonoids structurally similar to quercetin, including quercitrin, isoquercitrin, taxifolin, hyperoside, isorhamnetin, avicularin, fisetin, 6-C-β-D-glucopyranosyl-(2S,3S)-(+)-3′, 4′, 5,7-tetrahydroxyflavanol (GTDF), and 3,3′, 4′, 5,7-pentahydroxyflavone-6-C-β-D-glucopyranoside (PHFG).

## 3. Natural Polyphenols and Osteogenic Differentiation of MSCs

Extensive research has highlighted the remarkable potential of quercetin and structurally related flavonoids in promoting tissue regeneration, with a particular focus on their ability to induce osteogenic differentiation in MSCs (Table 1). These natural compounds have been shown to activate multiple signaling pathways and molecular mechanisms that contribute to bone formation and remodeling. In the following sections, we will explore the specific mechanisms of action underlying the osteogenic effects of quercetin, quercitrin, isoquercitrin, dihydroquercetin, hyperoside, isorhamnetin, avicularin, fisetin, GTDF, and PHFG, providing a comprehensive overview of their roles in enhancing MSC differentiation and bone regeneration.

### 3.1. Quercetin

Quercetin is a bioflavonoid present in a variety of vegetables and fruits, including capers, onions, lettuce, asparagus, green tea, apples, and berries. Extensive research has demonstrated that quercetin exhibits a broad spectrum of therapeutic effects, such as anti-allergic, antimicrobial, anti-diabetic, neuroprotective, cardioprotective, and hepatoprotective activities, underscoring its potential as a valuable pharmaceutical agent [22–27]. Moreover, quercetin possesses significant antioxidative properties, which are primarily attributed to the presence of five hydroxyl groups in its molecular structure [28, 29]. In this context, an animal study investigated the effects of quercetin on oxidative stress reduction and fetal ossification, highlighting its potential to mitigate the risk of bone dysfunction later in adulthood [30]. Similarly, quercetin has been reported to promote osteogenesis and exert protective effects against osteoporosis in ovariectomized rat models [31–34].

Numerous studies have documented the antioxidant effects of quercetin on BM-MSCs. Specifically, quercetin has been shown to enhance the expression of the antioxidant enzymes superoxide dismutase 1 (SOD1) and superoxide dismutase 2 (SOD2) in BM-MSCs [35]. Additionally, research has demonstrated that quercetin increases the survival of BM-MSCs under oxidative stress by preventing cell death mechanisms, such as apoptosis and ferroptosis. Furthermore, quercetin promotes the proliferation of these cells, supporting their regenerative potential [36–38]. Beside considerable effects on the survival and proliferation of BM-MSCs, quercetin induces osteogenic differentiation by modulating various cell signaling pathways. Specifically, it inhibits osteoclastogenesis and promotes osteoblastogenesis, leading to enhanced bone formation and reduced bone breakdown. Additionally, quercetin inhibits adipogenic differentiation in BM-MSCs [35, 38, 39]. This ability is particularly significant, as studies have shown that BM-MSCs from osteoporosis patients exhibit reduced osteogenic capacity and increased adipogenic differentiation [2]. Therefore, the potential of quercetin to both inhibit adipogenesis and induce osteogenesis in BM-MSCs makes it a promising therapeutic candidate for bone regeneration.

Investigating the molecular mechanisms underlying the effects of quercetin on BM-MSCs revealed that it increases the phosphorylation of AKT, PI3K, and mTOR, as well as the expression of SIRT1 through the phosphorylation of AMPKC. Furthermore, quercetin activates the ERK, p38, Wnt/β-catenin, and BMP signaling pathways, leading to the survival, proliferation and differentiation of BM-MSCs [35, 37, 40, 41]. Additionally, quercetin can induce osteogenic differentiation of BM-MSCs by inhibiting TNF-α/NF-κB and RANKL signaling pathways [39, 42]. Interestingly, quercetin exhibits phytoestrogen-like activity, allowing it to activate estrogen signaling pathway within BM-MSCs [43]. This is particularly significant, as estrogen receptor activation is known to suppress the function of RANKL, a critical regulator of osteoclastogenesis. By inhibiting this osteoclast-promoting factor, quercetin can effectively curb bone resorption and maintain bone homeostasis. Furthermore, estrogen signaling stimulated by quercetin also activates the Wnt/β-catenin pathway, which plays a pivotal role in driving osteogenic differentiation. Additionally, estrogen can activate BMP signaling to promote the maturation of pre-osteoblasts into fully functional osteoblasts, rather than directing BM-MSCs down the adipogenic lineage [39].

Besides its effects on receptors and proteins involved in cell signaling, quercetin modulates noncoding RNAs, specifically miR206, which subsequently increases the expression of connexin 43 in BM-MSCs [44]. Similarly, during the process of quercetin-induced differentiation in BM-MSCs, the expression of miR625-5P is reduced [45]. Conversely, the expression of H19 lncRNA increases during quercetin-induced differentiation in BM-MSCs. Studies have shown that the expression of H19, which stimulates osteogenic differentiation by activating the Wnt/β-catenin pathway, is inhibited in patients with osteoporosis and bone defects [46, 47]. Furthermore, H19 has been shown to downregulate several microRNAs, including miR-140-5p and miR-149, by which enhancing osteogenic differentiation in BM-MSCs [47–49].

Quercetin also acts as a senolytic agent that selectively removes senescent cells. Senescence is a cell fate characterized by irreversible replication arrest, resistance to apoptosis, and increased metabolic activity. Emergence of senescent cells in the bone microenvironment inhibits osteogenesis while promoting adipogenesis via secretion of senescence-associated secretory phenotype (SASP), which inhibits osteogenesis and promotes adipogenesis in BM-MSCs, contributing to the development of osteoporosis [50, 51]. In a study investigating the senolytic activity of quercetin, it was found that quercetin treatment effectively removed senescent bone marrow cells and enhanced the proliferation of BM-MSCs. This resulted in increased bone formation potential and decreased adipogenic potential [50]. A recent 2025 study revealed a novel mechanism by which quercetin restores osteogenic differentiation in senescent BM-MSCs. The research demonstrated that heterochromatin destabilization leads to the release of repetitive genomic elements (REs), resulting in the accumulation of double-stranded RNA (dsRNA) within the cytoplasm. This dsRNA activates the RIG-I pathway, triggering innate immune responses, chronic inflammation, accelerated cellular aging, and impaired bone formation. Quercetin mitigates these effects by stabilizing heterochromatin, preventing RE release, and inhibiting the dsRNA/RIG-I signaling cascade. Through these actions, quercetin reduces cellular senescence and restores the capacity of cells for bone regeneration [52].

Emerging studies have also documented the osteogenic differentiation of adipose-derived MSCs (AD-MSCs) following the administration of quercetin. It was found that quercetin enhanced the expression of OSX, Runx2, BMP-2, Col-1, Opn, and Occn and increased ERK phosphorylation in murine AD-MSCs [53, 54]. Likewise, quercetin induced osteogenesis in a study conducted on umbilical cord MSCs [55].

### 3.2. Quercitrin

The osteogenic induction effect of quercitrin has been reported in a few studies, as this flavonoid induced osteoblastogenesis and inhibited osteoclastogenesis in pre-osteoblasts [56]. Additionally, in a study utilizing quercitrin as a coating on titanium implants in the rabbit tibia model, significant reduction in osteoclastogenesis-associated genes, including Trap, CalcR, Ctsk, H + ATPase, and MMP9 was reported [57]. In another attempt, quercitrin has been investigated for its potential in treating periodontal disease, a condition characterized by gum infection and inflammation, leading to bone and tooth loss. In a laboratory model using human gingival fibroblasts and MSCs, quercitrin reduced the release of inflammatory mediator PGE2 and restored impaired collagen metabolism in fibroblasts. Additionally, it increased the activity and mineralization of alkaline phosphatase in MSCs, promoting their differentiation towards osteoblasts [58].

### 3.3. Isoquercitrin

Isoquercitrin (3-O-β-D-galactopyranoside) has the potential to induce osteogenic differentiation in BM-MSCs via upregulating the expression of transcription factors Runx2, bone sialoprotein (BSP) and activator 6 (ATF6). This flavonoid has also shown promising effects in vivo, as oral administration of isoquercitrin significantly increased osteogenesis via enhancing BMP2 Runx2, BSP, and ATF6 expression and increasing ALP activity and mineral deposition [59]. In a study conducted on human BM-MSCs, it was reported that isoquercitrin increased cell proliferation, enhanced ALP activity and extracellular mineralization, and upregulated RUNX2 and osteocalcin via activating Wnt/BMP signaling and modulating MAPK-regulated PPARγ activity [60]. It has also been shown that inhibiting the expression of Runx2 in osteoblasts by siRNA or adding noggin to the BM-MSC culture medium reduced the osteogenesis-inducing effects of isoquercitrin [61]. A recent study demonstrated that isoquercitrin inhibits osteoclastogenesis and prevents bone loss by activating the Nrf2-mediated pathway and suppressing ROS-induced NF-κB signaling. This flavonoid reduces RANKL-induced ROS production and downregulates NF-κB expression via the Nrf2-dependent mechanism, exhibiting significant anti-osteoclastogenic and bone-protective effects in both in vitro and in vivo models. This newly uncovered mechanism highlights the antioxidant and anti-inflammatory properties of isoquercitrin, positioning it as a promising agent for preventing bone resorption and treating rheumatoid arthritis [62].

### 3.4. Dihydroquercetin

Dihydroquercetin, also known as Taxifolin, is capable of inducing osteoblastogenesis, while inhibiting osteoclast differentiation [56]. During the osteogenesis induction, dihydroquercetin inhibits the activation of NF-κB signaling pathway caused by TNF-α in human BM-MSCs [63]. Dihydroquercetin also inhibited osteoclastogenesis and prevented bone resorption in a mouse calvarial osteolysis model [64]. Investigating effects of dihydroquercetin on diabetic rat models with periodontitis revealed downregulation of RANKL but induction of BMP-2, ALP, collagen type I, and OCN [65]. It has also been reported that dihydroquercetin induces protective and antiapoptotic effects on dental pulp MSCs [66].

### 3.5. Hyperside

Hyperoside is another flavonoid that increases ALP activity and calcium nodule formation via inhibiting ROS/JNK pathway [67]. In a study investigating the effect of hyperoside on periodontitis disease models, increased proliferation and osteogenesis induction, as well as NF-κB signaling activity were reported in rat BM-MSCs. In addition, improved alveolar bone resorption, relieved inflammatory infiltrates, increased regular arrangement of collagen fibers, and increased bone differentiation were observed upon administration of hyperoside [68].

### 3.6. Isorhamnetin, Avicularin, and Fisetin

Isorhamnetin suppresses RANKL-induced ROS generation, which mediates osteoclastogenesis. Additionally, isorhamnetin inhibites the formation and function of osteoclasts by inhibiting the activation of MAPK, NF-κB, and AKT signaling pathways in bone marrow macrophages. In a study injecting isorhamnetin to osteoarthritis mouse models, osteoclast overactivity and cartilage apoptosis were inhibited [69]. Similarly, avicularin has the potential to inhibit the activation and resorptive activity of osteoclasts and increase bone mineral density [70].

Recent research on flavonoids has revealed that, despite their structural similarity, fisetin and quercetin exert opposite effects on the differentiation of hMSCs. Fisetin strongly inhibits osteogenic differentiation, as evidenced by reduced mineralization and downregulation of osteogenic genes, such as *COL1A1* and *OCN*, while promotes adipogenic differentiation by increasing lipid accumulation and upregulating adipogenic markers like PPARγ and ADIPOQ. In the same study, a similar dose of quercetin had no significant effect on osteogenic differentiation but effectively suppresses adipogenic differentiation by downregulating genes involved in this process. These findings highlight how subtle structural differences between flavonoids can lead to markedly different influences on stem cell fate decisions [71].

### 3.7. GTDF and PHFG

GTDF and PHFG are quercetin C-glucoside compounds with osteogenesis-inducing effects. GTDF stimulates the proliferation, survival, and differentiation of osteoblasts by activating the aryl hydrocarbon receptor, without affecting osteoclastic or adipocyte differentiation. In vivo, GTDF promoted peak bone accrual in parameters in the appendicular skeleton, including increased longitudinal growth, bone mineral density, bone formation rate, cortical deposition, and overall bone strength [72]. Likewise, oral administration of PHFG in growing mice increased bone marrow osteoprogenitors, bone mineral density, bone formation speed, and cerebral cortex deposition. In addition, PHFG improved the speed of bone formation and trabecular microarchitecture in osteopenic mice [73].

### 3.8. Quercetin Complexes

Several quercetin complexes have been synthesized and evaluated for their ability to promote osteogenesis. For instance, a complex of vanadium and quercetin [VO(Quer) (2) EtOH] (n) (QuerVO) stimulated the production of type I collagen and activated ERK pathway in vitro [74]. In addition, copper (II) quercetin Cu + Q complex, quercetin–copper, (II)-phenanthroline [Cu + Q(PHt)], and quercetin-Cu (II)-neocuproin [Cu + Q(Neo)], induced osteogenesis and angiogenesis, increased calcium deposition, enhanced ALP activity, and upregulated Runx2 and collagen type 1 expression [75]. Furthermore, a novel strontium–quercetin complex [(C15H7O7)Sr_2_]·6(H_2_O) was investigated both in vitro and in animal models. This compound not only boosted ALP activity and extracellular matrix mineralization in pre-osteoblast cells but also significantly promoted bone regeneration in a mouse periapical defect model within 7 days. The synergistic osteogenic, antioxidant, and anti-inflammatory properties of strontium combined with quercetin resulted in superior support for bone differentiation and repair compared to conventional strontium compounds, positioning this complex as a promising and safe therapeutic candidate for bone diseases and defect healing [76].

## 4. Discussion

Overcoming the debilitating consequences of bone defects requires the development of innovative strategies that effectively restore bone integrity and function. While MSCs hold great promise for bone regeneration due to their differentiation potential and paracrine effects, optimal bone repair demands the integration of naturally derived, biocompatible osteogenic factors that can synergize with cellular therapies. Among natural polyphenols, quercetin has emerged as a particularly potent bioactive agent with multifaceted effects on bone tissue repair, as summarized in Figure 1. It orchestrates the activation of multiple key signaling pathways—including AMPK/SIRT1, Wnt/β-catenin, MAPK (ERK and p38), AKT/PI3K/mTOR, and BMP—that collectively promote osteogenic differentiation and enhance MSC proliferation. Simultaneously, quercetin inhibits bone resorption by suppressing pro-inflammatory cascades, such as TNF-α/NF-κB and RANKL signaling. Beyond protein pathways, quercetin modulates several noncoding RNAs (miR-206, miR-625-5p, miR-675, miR-149, miR-140-5p) and lncRNA H19, which further activate Wnt/β-catenin signaling, amplifying osteogenesis. Additionally, quercetin influences the expression of osteogenic and angiogenic factors including SATB2, estrogen receptors α and β, VEGF, angiopoietin-1, and connexin 43, thereby stimulating angiogenesis, reducing oxidative stress, and facilitating bone tissue regeneration. The molecule's senolytic properties also contribute to reducing age-related osteoporosis, highlighting its broad therapeutic potential. Studies investigating quercetin complexes with vanadyl (IV), copper (II), and strontium ions have reported enhanced bone repair effects, further emphasizing the benefits of combinatorial approaches.

However, the clinical application of quercetin and similar natural polyphenols is challenged by their limited solubility, stability, and bioavailability when administered alone. To overcome these barriers, advanced nanoformulations and topical drug delivery systems have been developed to improve cellular uptake and target action at sites of bone defects, thereby accelerating bone healing. As detailed in Table 2, recent advances demonstrate that incorporating natural flavonoids into biomaterial scaffolds and nanostructures—including electrospun nanofibers, hydrogels, microspheres, nanoparticles, and 3D-printed scaffolds—significantly enhances osteogenesis and bone tissue regeneration. These scaffolds provide controlled release of quercetin, creating a bioactive and therapeutically stable microenvironment that supports MSC growth and differentiation toward osteoblasts by upregulating critical transcription factors, such as RUNX2 and Osterix and activating BMP and Wnt/β-catenin pathways. The scaffolds' antioxidant and anti-inflammatory properties reduce oxidative stress and inflammation at injury sites, further promoting healing. Moreover, mineral nanomaterials like hydroxyapatite, silicates, and bioglass incorporated into these constructs improve mechanical strength and stimulate angiogenesis via increased VEGF expression. This synergy not only enhances new bone formation but also promotes bone remodeling by inhibiting osteoclast activity and bone resorption. In vitro and in vivo studies confirm that such combinatorial scaffolds outperform single-agent treatments, significantly improving healing outcomes in critical-sized bone defects and osteoporotic models [77].

Despite these encouraging outcomes, several important limitations temper the current understanding and clinical translation of these combinatorial therapies. First, there is potential publication bias, as positive results are more often reported, which may lead to an overestimation of therapeutic efficacy. Additionally, considerable heterogeneity exists across studies, including variations in scaffold fabrication methods, quercetin dosing regimens, animal models, and outcome measures. This diversity complicates direct comparisons and meta-analyses, thereby obscuring consistent mechanistic conclusions and therapeutic guidelines. Moreover, most mechanistic insights and efficacy data are derived from preclinical animal models that may not fully recapitulate human bone biology and pathology. Critical aspects, such as long-term safety profiles, optimal dosing ranges, scaffold degradation kinetics, and potential immunogenic or chronic toxic effects, remain insufficiently characterized.

To address these gaps and facilitate bridging between preclinical and clinical translational research, future research should prioritize specific study designs that include dose–response trials aimed at identifying the optimal concentration and release kinetics of quercetin within various scaffold materials, as well as long-term safety evaluations to monitor scaffold degradation, immune responses, and systemic toxicity over extended periods. In addition, rigorous head-to-head comparative studies in large-animal models are needed to thoroughly assess the efficacy of combinatorial scaffolds compared to monotherapy or MSC treatment alone. Furthermore, in-depth investigation of MSC fate, scaffold integration, and immunomodulatory effects at the injury site is essential, alongside efforts to standardize outcome measures to improve reproducibility and comparability across laboratories. Ultimately, well-designed clinical trials are crucial to establish evidence-based protocols for scaffold design, degradation rates, dosing regimens, and patient-specific therapies. By addressing publication bias and study heterogeneity through transparent reporting and systematic comparisons, future work can validate and optimize the synergistic benefits of combining quercetin with MSCs and biomaterial scaffolds. Only through such comprehensive and systematic investigations can these promising therapies be translated into safe, effective, and innovative treatments for complex bone defects.

## 5. Conclusion

Extensive preclinical evidence supports the osteogenic and bone-protective effects of natural polyphenols like quercetin, yet clinical application remains limited by poor solubility, stability, and bioavailability. Variability in study designs, including differences in delivery systems, dosing, and animal models, along with potential publication bias, complicates interpretation and translation. Future research should prioritize optimizing targeted delivery methods, conducting dose–response and long-term safety studies, and performing comparative evaluations in large-animal models to clarify the benefits of combining polyphenols with MSCs and biomaterial scaffolds. Mechanistic studies focusing on key pathways, such as Wnt, BMP, and RANKL/OPG, as well as the rejuvenation of senescent stem cells, will further refine therapeutic strategies. Additionally, standardizing outcome measures and promoting transparent reporting are essential for enhancing reproducibility. Importantly, well-designed clinical trials incorporating these considerations are crucial to validate and translate the synergistic potential of polyphenols combined with MSCs and biomaterial scaffolds, paving the way for effective bone regeneration therapies.

## Figures and Tables

**Table 1:** A summary of studies using polyphenols for osteogenic differentiation of MSCs.

Compound	Effects	Mechanism	Type of study	Reference
Quercetin	Promoted osteogenic differentiation, induced antioxidant responses	Activated AMPK/SIRT1 signaling pathway	In vitro	[35]
Quercetin	Protection from oxidative stress via ferroptosis	Reduced ROS, induced ferroptosis, upregulated antioxidant enzymes	In vitro	[40]
Quercetin	Stimulated differentiation through estrogen receptor-mediated pathway	Upregulated estrogen receptor alpha and beta downstream genes	In vitro	[37]
Quercetin	Promoted osteogenic differentiation, angiogenic factor secretion	Upregulated VEGF, ANG-1, promoted angiogenesis and osteogenesis	In vitro	[38]
Quercetin	Improved bone formation, increased osteogenic markers	Reduced senescent cells, improved BMSC function	In vitro	[39]
Quercetin	Increased bone formation in the mid-palatal suture	Promoted bone formation	In vitro	[41]
Quercetin	Promoted osteogenesis	Synergistic effects of quercetin and 3D-printed scaffolds	In vitro	[42]
Quercetin	Stimulated osteogenic differentiation	Upregulated miRNA-206 and connexin 43	In vitro	[44]
Quercetin	Promoted proliferation, osteogenic differentiation	Upregulated H19, miR-625-5p, activated Wnt/β-catenin pathway	In vitro	[45]
Quercetin	Activated Wnt signaling, promoted osteoblast differentiation	Functions as competing endogenous RNA binding to miR-675	In vitro	[46]
Quercetin	Promoted osteogenic differentiation	Upregulated H19, miR-675, activated Wnt/β-catenin pathway	In vitro	[47]
Quercetin	Stimulated osteogenic differentiation	Upregulated miR-149, SDF-1, activated Wnt/β-catenin pathway	In vitro	[48]
Quercetin	Promoted osteogenic differentiation	Upregulated miR-140-5p, SATB2, activated Wnt/β-catenin pathway	In vitro	[49]
Quercetin	Regulated proliferation potential and lineage commitment	Wnt signaling	In vitro	[55]
Quercetin	Promoted cell proliferation, osteogenic differentiation, and angiogenic factor secretion; inhibited osteoclastogenesis	MAPK and AKT signaling pathways	In vitro In vivo	[53]
Quercetin	Promoted osteogenic differentiation and survival of MSCs, inhibited osteoclastogenesis	Activation of Wnt/β-catenin, BMP, ERK, p38, phosphorylation of AKT, PI3K, mTOR, AMPK pathways; inhibition of TNFα/NF-κB and RANKL	In vitro In vivo	[52]
Quercitrin	Promoted osteoblast differentiation in MC3T3-E1 cells, inhibited osteoclastogenesis	Increased expression of bone sialoprotein and osteocalcin, decreased RANKL gene expression	In vitro	[56]
Quercitrin	Reduced osteoclast activity	Decreased osteoclast activity	In vitro In vivo	[57]
Quercitrin	Promoted proliferation and differentiation of cells	Potential role in periodontal regeneration	In vitro	[58]
Isoquercitrin	Promoted cell proliferation, ALP activity, and mineral deposition	Upregulated Runx2, BSP, and ATF6	In vitro In vivo	[59]
Isoquercitrin	Inhibited adipogenic differentiation, enhanced osteoblastogenic differentiation	Decreased the expression of adipogenic marker genes, increased ALP activity and upregulated the expression of osteoblastogenic markers	in vitro	[60]
Isoquercitrin	Increased viability and mineralization, upregulated osteogenic marker genes	RUNX2 or BMP signaling pathway	In vitro	[61]
Isoquercitrin	Inhibited osteoclastogenesis and reduced bone loss (in rheumatoid arthritis model)	Activation of Nrf2 pathway and inhibition of RANKL-induced ROS-NF-κB pathway, reduction of ROS production, inhibition of NF-κB expression	In vitro In vivo	[62]
Taxifolin	Stimulated osteoblast differentiation, inhibited osteoclastogenesis	Osteogenic and anti-osteoclastic activity	In vitro	[56]
Taxifolin	Enhanced osteogenic differentiation	Partially via NF-κB pathway	In vitro	[63]
Taxifolin	Inhibited RANKL-induced osteoclastogenesis, prevented LPS-induced bone loss	Anti-osteoclastic activity	In vitro in vivo	[65]
Taxifolin	Increased bone formation, reduced apoptosis	Anti-inflammatory and antioxidant activity	In vivo	[64]
Taxifolin	Protected from hypoxia-induced apoptosis and inflammation-induced damage	Anti-inflammatory and antioxidant activity	In vitro	[66]
Hyperside	Inhibited osteoblast apoptosis, increased cell viability	Targeting NOX4 to inhibit ROS accumulation and activate JNK pathway	In vitro	[67]
Hyperside	Promoted osteogenic differentiation, ameliorated periodontitis	Activated NF-κB pathway	In vivo	[68]
Isorhamnetin	Inhibited osteoclastogenesis, protected chondrocytes from oxidative stress-induced apoptosis	Modulated ROS homeostasis	In vitro In vivo	[69]
Avicularin	Inhibited osteoclastogenesis	Inhibited NF-κB signaling pathway	In vivo	[70]
Fisetin	Inhibited osteogenic differentiation, proliferation, and migration of MSCs	Downregulation of YAP activity and its binding to TEAD, decreased expression of osteogenic genes, upregulation of adipogenesis	In vitro In vivo	[71]
GTDF	Stimulated proliferation, survival, and differentiation, increased bone mineral density, and cortical bone strength	Aryl hydrocarbon receptor (AhR) mediated	In vitro In vivo	[72]
PHFG	Stimulated differentiation, increased bone mineral density, bone formation rate, and cortical deposition	Direct stimulatory effect on osteoprogenitors	In vitro In vivo	[73]
Quercetin vanadyl (IV) complexes	Exhibited antitumoral activity, stimulated osteogenic differentiation	Antioxidant and anti-inflammatory properties	In vitro	[74]
Quercetin copper(II) complexes	Exhibited osteogenic and angiogenic properties	Copper ions and quercetin interacted to regulate osteogenesis and angiogenesis	In vitro	[75]
Strontium–quercetin complex	Promoted osteogenic differentiation and extracellular matrix mineralization, bone defect repair	Increased alkaline phosphatase activity and matrix mineralization, direct effects on pre-osteoblastic cells	In vitro In vivo	[76]

**Table 2:** Quercetin based biomaterials for bone regeneration.

Compound/biomaterial complex	Effects on osteogenesis and stem cells	Mechanism	Type of study	Reference
Quercetin/collagen/hydroxyapatite sponge (freeze-dried)	Promoted highest proliferation and osteogenic differentiation of BMSCs	Enhanced osteogenic gene expression, mimics ECM, provides controlled release	In vitro, in vivo	[78]
Silk fibroin/hydroxyapatite/quercetin scaffold (freeze-dried)	Enhanced osteogenesis, 80% bone volume recovery in defect model, upregulated bone genes in BMSCs	Mechanical support, upregulation of bone genes, BMSC differentiation	In vitro, in vivo	[79]
PLGA/MgO/quercetin nanofiber (electrospinning)	Enhanced proliferation, migration, osteogenic differentiation, and angiogenesis of BMSCs	Porous structure, Wnt/β-catenin activation, angiogenic factor upregulation	In vitro, in vivo	[80]
Zn (quercetin) (phenanthroline) in PCL/gelatin nanofiber (electrospinning)	Induced osteoblast differentiation, stimulated osteogenic markers and angiogenesis in BMSCs	Increased ALP, calcium deposition, enhanced osteogenic gene expression	In vitro, in vivo	[81]
Quercetin-coupled Mg-doped calcium silicate ceramic (electrospinning)	Enhanced proliferation, collagen, ALP activity in BMSCs, reduced bacterial adhesion	Synergistic antibacterial and osteogenic effects, optimal quercetin concentration	In vitro	[82]
Quercetin + CDHA (3D printing)	Improved mechanical properties, steady quercetin release, enhanced bone formation, suppressed resorption, affected osteoblasts/osteoclasts	Modulation of osteoblast/osteoclast activity, sustained release	In vitro	[83]
Quercetin/polydopamine-poly (l-lactide; 3D printing)	Enhanced proliferation, ALP activity, calcium nodules, osteogenic gene/protein expression in stem cells	Sustainable release, upregulation of osteogenic markers	In vitro	[84]
Quercetin + β-TCP-PCL + KCl (3D printing)	Biphasic quercetin release, improved cell attachment and proliferation of osteoblasts	Tunable degradation, sustained delivery, promoted osteoblast growth	In vitro	[85]
Quercetin/nano-hydroxyapatite/decellularized scaffold	Improved BMSC growth, induced bone formation, promoted osteoblast differentiation	Biomimetic environment, enhanced osteogenic differentiation	In vitro	[86]
Chitosan/collagen + β-TCP + quercetin hydrogel	Porous, sustained quercetin release, noncytotoxic, supported encapsulation, and differentiation of stem cells	Bioactive microenvironment, controlled drug delivery	In vitro	[87]
Hydroxyapatite/alginate/quercetin microsphere	Induced osteogenesis, long-term release, promoted tissue repair, differentiation of progenitor cells to osteoblasts	Osteoblast progenitor differentiation, sustained release	In vitro	[88]
PLGA microsphere + quercetin	Increased ALP, Runx2, enhanced osteogenesis, long-term release in stem cell spheroids	Osteogenic gene upregulation, controlled release	In vitro	[89]
HA bioceramic microsphere + quercetin	Promoted osteogenesis and angiogenesis in OVX rat BMSCs, enhanced new bone and vessel formation	Sustained release, enhanced bone and vessel formation	In vitro, in vivo	[53]
Nanoscale bioglass microspheres/hydrogel + quercetin	Promoted osteogenic differentiation of orofacial MSCs and periodontal bone repair	Reduced oxidative stress, m6A/Per1 pathway modulation	In vitro	[90]
β-TCP granules + dasatinib/quercetin (senolytics)	Enhanced bone formation, reduced ROS, modulated senescence, improved BMSC function	Cleared senescent cells, improved bone-forming ability	In vivo	[91]
Quercetin liposome (bone affinity peptide)	Cleared old cells, increased BMSC proliferation, strengthened bone formation in aging models	Targeted delivery, senolytic effect, restored BMSC function	In vitro, in vivo	[92]
Quercetin-loaded phytosome nanoparticles	Improved bone parameters superior to free quercetin, promoted osteogenesis in stem cells	High encapsulation, enhanced bioavailability, hormone replacement effect	In vivo	[93]
Quercetin-loaded mesoporous bioactive glass nanoparticle	Promoted bone regeneration, regulated immune microenvironment, enhanced osteogenic differentiation of stem cells	Sustained release, osteoimmune modulation	In vivo	[94]

## Data Availability

The data sharing is not applicable to this article as no datasets were generated or analyzed during the current study.

## Nomenclature

AD-MSCs: Adipose-derived mesenchymal stem cells
ATF6: Activating transcription factor 6
ALP: Alkaline phosphatase
AMPK: AMP-activated protein kinase
BM-MSCs: Bone marrow mesenchymal stem cells
BMP2: Bone morphogenetic protein 2
BSP: Bone sialoprotein
Col-1: Collagen type I
dsRNA: Double-stranded RNA
ERK: Extracellular signal-regulated kinase
HPLC: High-performance liquid chromatography
lncRNA: Long noncoding RNA
MAE: Microwave-assisted extraction
MAPK: Mitogen-activated protein kinase
miR: MicroRNA
mTOR: Mechanistic target of rapamycin
MSCs: Mesenchymal stem cells
NF-κB: Nuclear factor kappa-light-chain-enhancer of activated B cells
Nrf2: Nuclear factor erythroid 2–related factor 2
Opn: Osteopontin
OSX: Osterix
PI3K: Phosphoinositide 3-kinase
REs: Repetitive genomic elements
RANKL: Receptor activator of nuclear factor kappa-B ligand
ROS: Reactive oxygen species
Runx2: Runt-related transcription factor 2
SASP: Senescence-associated secretory phenotype
SFE: Supercritical fluid extraction
SIRT1: Sirtuin 1
SMAD: SMAD proteins
SOD1: Superoxide dismutase 1
SOD2: Superoxide dismutase 2
SPE: Solid-phase extraction
TNF-α: Tumor necrosis factor alpha
UAE: Ultrasound-assisted extraction
UC-MSCs: Umbilical cord mesenchymal stem cells
Wnt: Wingless-related integration site
AKT: Protein kinase B.
